# Genome-Wide Characterization of RNA Editing in Chicken Embryos Reveals Common Features among Vertebrates

**DOI:** 10.1371/journal.pone.0126776

**Published:** 2015-05-29

**Authors:** Laure Frésard, Sophie Leroux, Pierre-François Roux, Christophe Klopp, Stéphane Fabre, Diane Esquerré, Patrice Dehais, Anis Djari, David Gourichon, Sandrine Lagarrigue, Frédérique Pitel

**Affiliations:** 1 INRA, Génétique, Physiologie et Systèmes d’Elevage, Castanet-Tolosan, France; 2 Université de Toulouse, INP, ENSAT, Génétique, Physiologie et Systèmes d’Elevage, Castanet-Tolosan, France; 3 Université de Toulouse, INP, ENVT, Génétique, Physiologie et Systèmes d’Elevage, Toulouse, France; 4 Agrocampus Ouest, Physiologie, Environnement et Génétique pour l'Animal et les Systèmes d'Élevage, Rennes, France; 5 INRA, Physiologie, Environnement et Génétique pour l'Animal et les Systèmes d'Élevage, Rennes, France; 6 INRA, Sigenae Biométrie et Intelligence Artificielle, Castanet-Tolosan, France; 7 INRA, GeT-PlaGe Genotoul, Castanet-Tolosan, France; 8 INRA, Pôle d'Expérimentation Avicole de Tours, Nouzilly, France; University of North Carolina at Charlotte, UNITED STATES

## Abstract

RNA editing results in a post-transcriptional nucleotide change in the RNA sequence that creates an alternative nucleotide not present in the DNA sequence. This leads to a diversification of transcription products with potential functional consequences. Two nucleotide substitutions are mainly described in animals, from adenosine to inosine (A-to-I) and from cytidine to uridine (C-to-U). This phenomenon is described in more details in mammals, notably since the availability of next generation sequencing technologies allowing whole genome screening of RNA-DNA differences. The number of studies recording RNA editing in other vertebrates like chicken is still limited. We chose to use high throughput sequencing technologies to search for RNA editing in chicken, and to extend the knowledge of its conservation among vertebrates. We performed sequencing of RNA and DNA from 8 embryos. Being aware of common pitfalls inherent to sequence analyses that lead to false positive discovery, we stringently filtered our datasets and found fewer than 40 reliable candidates. Conservation of particular sites of RNA editing was attested by the presence of 3 edited sites previously detected in mammals. We then characterized editing levels for selected candidates in several tissues and at different time points, from 4.5 days of embryonic development to adults, and observed a clear tissue-specificity and a gradual increase of editing level with time. By characterizing the RNA editing landscape in chicken, our results highlight the extent of evolutionary conservation of this phenomenon within vertebrates, attest to its tissue and stage specificity and provide support of the absence of non A-to-I events from the chicken transcriptome.

## Introduction

A fascinating reality of the genome, with more and more empirical evidence, is that its biology is far more complex than previously thought. The rule "one gene has one DNA sequence leading to one mRNA translated into one protein", even if not (yet) an exception, is now well-known to have evolved in a vast field of other possibilities. Taking the example of the human genome, the number of genes, the percentage of the genome that is transcribed, the alternative transcripts count per gene, or the way their expression is regulated, are all characteristics for which knowledge is moving with an extraordinary pace. The ENCODE project brought a lot of data and analyses in this area [1]. Among transformations that RNA transcripts undergo during maturation, RNA editing is a phenomenon leading to differences between the final RNA sequence and the DNA region it was transcribed from. The term was first used by Benne *et al* in 1986 [2], and can now be defined, in a broad sense, as a nucleotide insertion, deletion or substitution in the RNA sequence, occurring in various types of RNA, from tRNA to mRNA, whether coding or not [3]. Substitutions comprise several types of modifications, the most common in vertebrates being the A-to-I conversion, catalyzed by the ADAR family enzymes (Adenosine Desaminase that Acts on RNA) [4] and leading to an A-to-G reading of the cDNA molecule [5, 6] and C-to-U conversion, catalyzed by the APOBEC enzyme [7, 8].

RNA editing is limited to eukaryotes, with a few exceptions (see [9] for review). It is observed in chloroplasts, widespread in mitochondria, and also found as a nuclear phenomenon in animals. It seems to have arisen through different mechanisms in different lineages, rather than being inherited from a common ancestor, and whether natural selection was involved in its evolution is still debated [9–11]. While RNA editing is more widely characterized in mammals, especially in human, mouse and rat [12–18], only a few studies have been performed in birds and these were targeting specific genes. The apolipoprotein B (APOB) RNA editing mechanism, well-known in mammals, seems to be absent from chicken [19] and zebra finch [20], which constitutes an argument in favor of the absence of the C-to-U editing phenomenon in these species. In chicken, the *CYFIP2* (cytoplasmic FMR1 interacting protein 2) and *FLNA* (filamin A) genes are edited in brain and liver [21], the splicing regulator *NOVA1* (Neuro-Oncological Ventral Antigen 1) is edited in the brain [22] and the *GABA*_*A*_ (gamma-Aminobutyric Acid Type A) Receptor, alpha3 subunit (*GABRA3*) is edited in the brain and the retina [23, 24]. But no genome-wide study in chicken is available. High-throughput RNA sequencing allows a deeper transcriptome analysis than previous technologies, including RNA editing through a genome-wide approach [25]. This has been performed on several species, including human and mouse [12, 13, 15, 26, 27] but never in avian species. The number of editing sites (detected as RDD: RNA-DNA Differences) observed in mammals varies widely between studies, even in the same tissues of the same species, and an increasing number of analyses point to the requirement of very careful bioinformatics procedures to limit technical artifacts [14, 15, 28–32].

To improve the available knowledge about the extent of RNA editing in chicken, we chose an approach without *a priori* knowledge by using DNA and RNA sequencing on the same samples through Next Generation Sequencing (NGS) technology of whole embryos. Our results support the fact that RNA editing seems to be limited to A-to-I conversions in chicken, shows strong tissue- and developmental-specificities and is conserved among vertebrates at specific coding sites.

## Materials and Methods

### Ethics Statement

Chickens were bred at INRA, UE1295 Pôle d’Expérimentation Avicole de Tours, F-37380 Nouzilly in accordance with European Union Guidelines for animal care, following the Council Directives 98/58/EC and 86/609/EEC. Animals were maintained under standard breeding conditions, being subjected to minimal disturbance. The farm is registered by the Ministry of Agriculture with the license number C37–175–1 for animal experimentation. The experiment was realised under authorization 37–002 delivered to D. Gourichon. Embryos were harvested for another study [33] after incubation in standard conditions.

### Tissues dataset

The material used in this study for the embryo sequences dataset was previously described [33] [SRA study accession number: SRP033603]. Briefly, two chicken lines were crossed, Line 6 [34] and Line R^-^ [35]. Twelve F1 were produced from 2 families: 8 embryos (embryonic day 4.5) and 4 adults from the same batch. Embryos were kept as a whole, while 3 adult tissues were harvested: brain, heart, and liver, after electronarcosis and immediate bleeding. Additional embryos were produced at embryonic days 4.5 (n = 8) and 15 (n = 8), from a cross between the same lines, and 3 embryonic tissues were harvested: brain, heart and liver. Genomic DNA and total RNA were concurrently extracted from the same samples of whole embryos or individual tissues (AllPrep DNA/RNA Mini Kit, Qiagen). RNA quality was measured by a BioAnalyzer (Agilent); all samples had a RIN (RNA Integrity Number) ≥ 9.9.

### Sequencing

#### RNA sequencing

Libraries with a mean insert size of 200bp were prepared following Illumina instructions for RNA-Seq analysis, by selecting polyA+ fragments (TruSeq RNA Sample Prep Kit) from each sample. Samples were tagged to allow subsequent identification, amplified by PCR and quantified by qPCR (Agilent QPCR Library Quantification Kit).

A total of 8 embryo libraries were sequenced (paired-ends, 100 bp) in triplicate after multiplexing, on an Illumina HiSeq 2000 sequencer (Illumina, TruSeq PE Cluster Kit v3, cBot and TruSeq SBS Kit v3) by randomizing their position in 6 different sequencing lanes.

#### DNA sequencing

DNA from 8 embryos was sequenced after multiplexing on 5 lanes of Illumina HiSeq 2000. Library preparation (mean insert size 328bp), DNA quantification and sequencing (paired-ends, 100bp) were performed according to the manufacturer’s instructions (TruSeq DNA Sample Prep Kit Illumina, Agilent QPCR Library Quantification Kit, TruSeq PE Cluster Kit v3 cBot TruSeq SBS Kit v3).

### Computational analyses

When not specified, analyses were performed with in-house Perl and R scripts.

All studied samples were aligned independently with regard to their sequencing lane, and then merged by individual before identification of RNA/DNA differences.

#### Genomic sequences analyses

Sequences were aligned to the current chicken genome assembly (Gallus gallus 4) using the BWA program version 0.7.0, option aln [36]. Sequences were then filtered on mapping quality (MAPQ≥30). SAMtools rmdup command was used to remove possible PCR duplicates.

#### PolyA RNA sequences analysis

Sequences were aligned with Tophat software version 2.0.5 on the chicken reference genome Galgal4 as described in [33].

Sequences mapping uniquely on the reference genome, without PCR duplicates and with a minimum mapping quality of 30, were selected.

#### Identification of RNA/DNA differences

Sequences were locally realigned and recalibrated before SNP detection with GATK software version 1.6.11 and BamUtil (bam recab command).

SAMtools software version 0.1.19 was used with mpileup utility to detect SNPs between DNA and RNA samples from each individual. We set a maximum coverage of 10,000 for each calling to take into account as many reads as possible in the calling. SNPs were detected independently on each biological replicate.

#### Editing detection

SNPs were analyzed from VCF files obtained from SAMtools mpileup detection. For each biological replicate, only variations where DNA was homozygous either for the reference allele or for the alternative allele, and where RNA was heterozygous, were kept.

Several successive filters were applied to consider a position as a putative RDD site. We first only considered positions with a sufficient depth, keeping only candidates presenting a minimum of 15 reads both in DNA and RNA alignments. To increase the likelihood of a site to be a true RDD position by avoiding a sample artefact, we set to 2 the number of biological replicates that must carry the same modification.

We then applied several filters inherent to technical bias due to high-throughput sequencing.

As there is an over-representation of mis-called SNP in read extremities [37–39], we discarded each RDD site for which the median position of the "edited" allele among reads overlapping them was in the 10 first or 10 last bases. In accordance with previous studies [28], we chose to consider only the distribution of the “edited” nucleotide position, to increase the stringency of the method.

The strand bias was also considered; to be kept, a RDD candidate must present a proportion of edited allele on the forward strand close to its proportion on reverse strand (delta≤0.5).

We checked the biallelic status of each selected candidate, a third allele being detected in less than 5% of cases was considered as a sequencing error.

An additional filter was applied to ensure that the alternative nucleotide frequency in DNA was null.

The functional consequence of each RDD in each transcript was predicted using the Ensembl Variant Effect Predictor (VEP) version 71 [40]. Non-coding splicing site regions were removed to take into account putative misaligned reads at these sites [31]. Then, positions belonging to homopolymers (n≥5) were removed because they may generate false positive candidates [13].

The chicken genome assembly still lacks several assembled regions, due to sequence assembly errors or missing fragments. A fragment detected as uniquely mapped may thus be present, with several polymorphisms, at genomic regions absent from the reference sequence, but present in the DNA reads from our samples. Therefore, a last filter was performed by searching the “editing site” (40 bp surrounding the candidate locus) in the DNA reads from samples thought to be edited. This pattern was searched with fuzznuc [41].

### Validation assays and editing characterization

#### Sanger sequencing

We first checked the homozygous status of RDD sites by Sanger sequencing of DNA. The 8 biological replicates were tested. Primers were designed using PyroMark Assay Design software to allow further cDNA pyrosequencing (S1 Table).

#### Pyrosequencing

RDD sites were tested on a Qiagen PyroMark Q24 sequencer. Primers were designed with PyroMark Assay Design software (S1 Table). PCR products were made using PyroMark PCR Kit (Qiagen). We performed the analyses through the PyroMark Q24 1.0.10 software with default analysis parameters.

Tissue and stage effects on the editing level were tested through an analysis of variance in a model taking into account tissues, stages and the interaction between tissues and stages for each tested candidate.

#### *In Silico* prediction of protein structure and function

To predict the putative effect of the editing conversions on protein structure and function, we used several bioinformatic tools: SIFT is based on sequence homology and the physical properties of amino acids (http://sift.bii.a-star.edu.sg/); PolyPhen2 uses physical and comparative considerations (http://genetics.bwh.harvard.edu/pph2/); MutationAssessor is based on evolutionary conservation of the affected amino acid in protein homologs (http://mutationassessor.org/); CHASM (computed using CRAVAT 3.0: http://www.cravat.us/) is based on the probability that a modification gives the cells a selective survival advantage; ProSMS predicts protein stability changes due to single amino acid modifications (http://babel.ucmp.umu.se/prosms/).

## Results

### Sequences analysis

DNA and RNA sequences were obtained from the same samples of chicken embryos. On average, 141,534,451 DNA reads and 65,302,559 RNA reads were aligned and analyzed for each embryo. The genome coverage reaches 93% for DNA reads and 22% for the RNA reads. A summary of DNA and RNA sequences aligned to the Galgal4 chicken assembly is presented in Table 1.

**Table 1:** Number of analyzed RNA and DNA sequences in the study (after alignment to Galgal4).

**Mean total number of reads (DNA)**	141,534,451
**Mean total number of reads (RNA)**	65,302,559
**Total number of reads (DNA)**	1,132,275,604
**Total number of reads (RNA)**	522,420,469
**Mean coverage (DNA)—min coverage 5 reads (% genome)**	93.1 ± 1.0
**Mean coverage (RNA)—min coverage 5 reads (% genome)**	22.3 ± 2.1
**Mean coverage (RNA)—min coverage 15 reads (% genome)**	16.4 ± 2.6

### Data filtering—detection of biases

**Fig 1:**
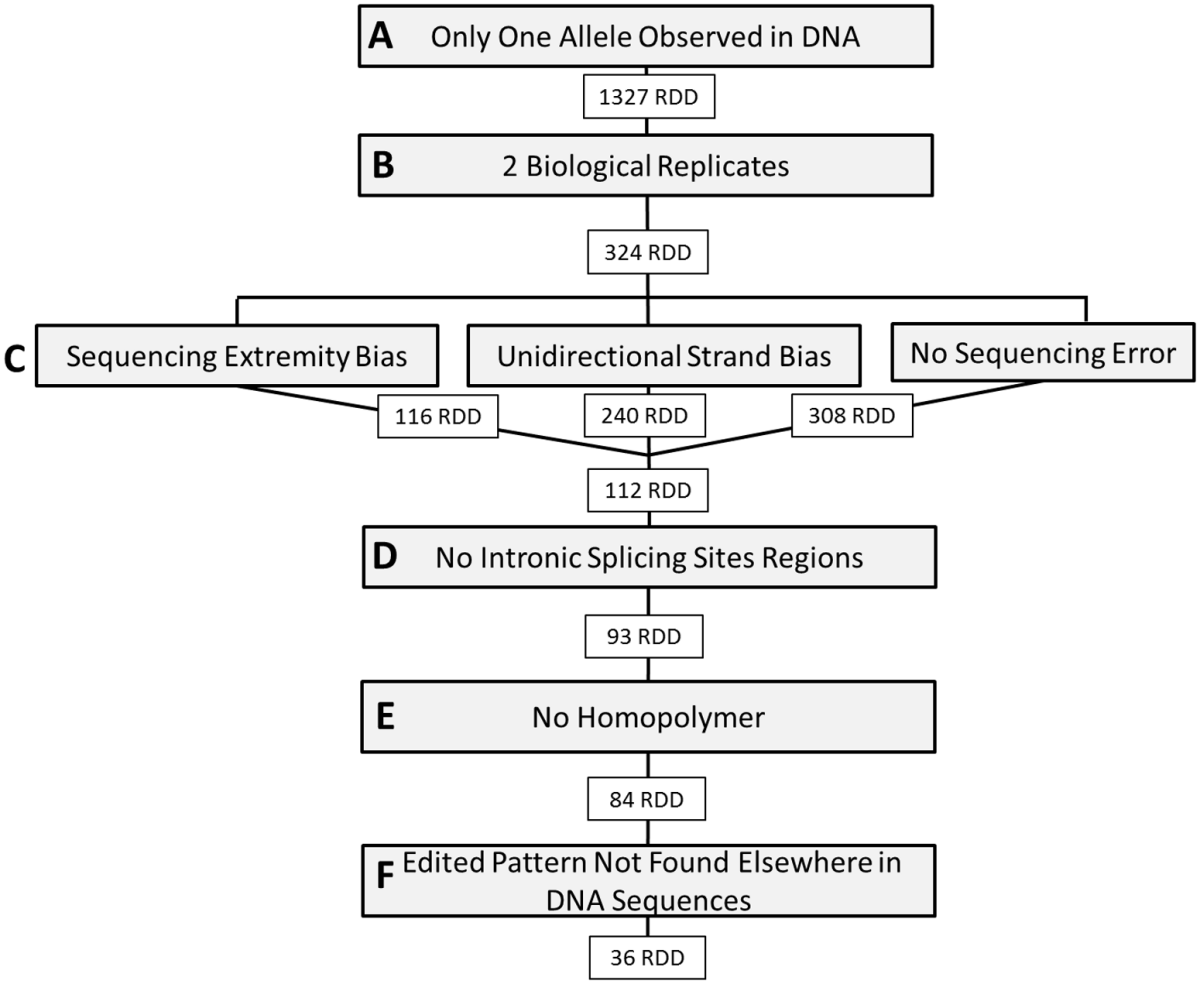
Number of RDD candidates obtained after each filter.

The first step was to detect RDD sites, *i*.*e*. positions homozygous in DNA and presenting an alternative sequence in RNA. To consider a position as a potential candidate, we fixed a minimum read-depth threshold of 15 both in DNA and RNA alignments for each embryo. We kept only candidates for which the alternative nucleotide frequency in DNA was null (Fig 1A). A total of 1,327 RDD sites met this criterion. The next filtering steps are aiming to avoid common pitfalls in sequences analysis thus increasing the robustness of the results (Fig 1). To avoid putative false positive due to an artifact present only in one sample, we only considered RDD sites detected in at least 2 biological replicates. We ended up with 324 RDD sites (Fig 1B).

It has previously been shown that polymorphisms overrepresented in read extremities are likely to be false positives [37–39]. In order to avoid this bias, we considered only RDD sites that were, in median, not in the 10% extremities of RNA reads overlapping them (Fig 1C). Two additional filters related to sequencing were applied: we removed candidates with an over-representation of one allele on one strand and discarded positions where more than one alternative nucleotide was found in proportions greater than 5% (Fig 1C). A total of 112 RDD sites passed these filters. We then removed candidates in splicing sites from non-coding regions (Fig 1D), and filtered for regions containing homopolymers (Fig 1E). A total of 84 candidates remained. We applied a last filter by removing candidates harboring the “edited” pattern in the genomic DNA reads (Fig 1F). The goal was here to take into account putative candidate regions for which the corresponding DNA reads were present in raw sequence data, but unmapped or not mapped to the same position as the "edited" RNA reads.

At the end of the analysis, we found 36 reliable RDD candidates (Table 2). A total of 17 chicken genes are potentially impacted by these RDD sites, knowing that one site can be associated with several genes and that we are probably missing non-annotated genes for candidates highlighted in intergenic regions. Interestingly, many of these candidates were organized in clusters, the 36 positions corresponding to 20 different genomic regions (Table 2). A total of 7 clusters, in 5 annotated genes and in 2 intergenic regions, could be counted, encompassing 12 to 1,439 bp. The distance between 2 clustered RDDs ranged from 3 to 807 bp, for a number of clusters comprised of between 2 and 5 detected sites.

**Table 2:** RDD candidates after filtering steps.

Chromosome	Position	DNA Nucleotide	RDD Nucleotide	Gene Name	Gene Short Name	Consequence (VEP analysis)	Number Of Edited Replicates	Mean Depth	Mean Frequency Of Edited Nucleotide
1	36367200	A	G	ENSGALG00000027799	*TMEM19*	3_prime_UTR_variant	2	25±2.83	0.27±0.11
1	74991790	T	C	ENSGALG00000014342	*TEAD4*	downstream_gene_variant, intron_variant	7	27.14±9.44	0.38±0.1
1	74992334	T	C	ENSGALG00000014342	*TEAD4*	downstream_gene_variant, intron_variant	3	38.33±12.34	0.46±0.07
1	74992422	T	C	ENSGALG00000014342	*TEAD4*	downstream_gene_variant, intron_variant	3	30.33±6.66	0.3±0.06
1	74993229	T	C	ENSGALG00000014342	*TEAD4*	downstream_gene_variant, intron_variant	3	25.33±7.51	0.39±0.06
**1**	**167109833**	**A**	**G**	**ENSGALG00000016980**	***COG3***	**missense_variant**	**4**	**100.25±32.8**	**0.47±0.08**
**2**	**86000822**	**T**	**C**	**ENSGALG00000013191**	***NDUFS6***	**upstream_gene_variant**	**5**	**73.2±19.64**	**0.42±0.06**
2	86000881	T	C	ENSGALG00000013191	*NDUFS6*	upstream_gene_variant	2	114.5±36.06	0.21±0
**2**	**86000926**	**T**	**C**	**ENSGALG00000013191**	***NDUFS6***	**upstream_gene_variant**	**5**	**113.2±64.58**	**0.48±0.06**
**2**	**86001360**	**T**	**C**	**ENSGALG00000013191**	***NDUFS6***	**upstream_gene_variant**	**4**	**61.75±24.92**	**0.26±0.06**
**2**	**86001370**	**T**	**C**	**ENSGALG00000013191**	***NDUFS6***	**upstream_gene_variant**	**5**	**55.8±26.13**	**0.43±0.09**
2	110994632	A	G	-		intergenic_variant	3	29.33±3.79	0.67±0.11
**3**	**2384093**	**A**	**G**	**-**	**-**	**intergenic_variant**	**5**	**21.8±6.26**	**0.28±0.13**
**3**	**2384105**	**A**	**G**	**-**		**intergenic_variant**	**2**	**23±9.9**	**0.34±0.06**
3	38271681	T	C	ENSGALG00000011013	*PCNXL2*	splice_region_variant, synonymous_variant	2	40±16.97	0.87±0.01
4	17996546	T	C	-		intergenic_variant	3	24.33±7.09	0.27±0.04
4	17999411	T	C	ENSGALG00000009128	*novel gene*	downstream_gene_variant	3	36±14.42	0.3±0.07
4	17999509	T	C	ENSGALG00000009128	*novel gene*	downstream_gene_variant	5	33±14.82	0.26±0.05
**4**	**20956439**	**A**	**G**	**ENSGALG00000009405**	***GRIA2***	**missense_variant, splice_region_variant**	**5**	**48±53.30**	**0.42±0.09**
**4**	**73361456**	**A**	**G**	**ENSGALG00000014395**	***DHX15***	**downstream_gene_variant**	**4**	**25.5±3.87**	**0.39±0.16**
6	15730446	A	G	ENSGALG00000005243	*PPP3CB*	intron_variant	2	18.5±4.95	0.38±0.25
**6**	**29787642**	**A**	**C**	**ENSGALG00000009427**	***TIAR***	**intron_variant**	**4**	**49.5±14.84**	**0.29±0.12**
6	34848568	A	G	ENSGALG00000010517	*TCTN3*	downstream_gene_variant	3	34±7	0.2±0.04
12	2800528	T	C	ENSGALG00000003738 ENSGALG00000003799	*EMC3 USP4*	downstream_gene_variant	3	29.33±10.97	0.55±0.19
12	2800601	T	C	ENSGALG00000003738 ENSGALG00000003799	*EMC3 USP4*	downstream_gene_variant	2	32.5±2.12	0.22±0.01
**13**	**931843**	**T**	**C**	**ENSGALG00000000946**	***PFDN1***	**intron_variant**	**2**	**19.5±0.71**	**0.28±0.1**
**13**	**931855**	**T**	**C**	**ENSGALG00000000946**	***PFDN1***	**intron_variant**	**3**	**22.67±4.73**	**0.65±0.09**
**13**	**931888**	**T**	**C**	**ENSGALG00000000946**	***PFDN1***	**intron_variant**	**2**	**23.5±2.12**	**0.28±0.06**
**13**	**10717577**	**T**	**C**	**ENSGALG00000003818**	***CYFIP2***	**missense_variant**	**2**	**40.5±17.68**	**0.27±0.08**
17	4705	T	C	ENSGALG00000014171	*novel gene*	intron_variant	3	45±6.56	0.18±0.03
22	5274	T	C	-	-	intergenic_variant	2	16±0	0.7±0.18
Z	27752047	A	G	-	-	intergenic_variant	3	29±4.58	0.23±0.06
Z	27752050	A	G	-	-	intergenic_variant	3	28±4	0.84±0.05
Z	27752217	A	G	-	-	intergenic_variant	2	31±4.21	0.57±0.01
Z	30809802	T	C	-	-	intergenic_variant	2	23±7.1	0.25±0.05
Z	30815599	T	C	ENSGALG00000005846	*MPDZ*	downstream_gene_variant	2	21.5±4.95	0.33±0.16

In bold, candidates tested for validation

### RDD types

We distinguished canonical RDD (A-to-G and C-to-T) from non-canonical RDD (other base changes). As the sequencing process was not strand-specific, the complement bases of canonical changes were also considered as canonical (*i*.*e*. T-to-C and G-to-A).

**Fig 2:**
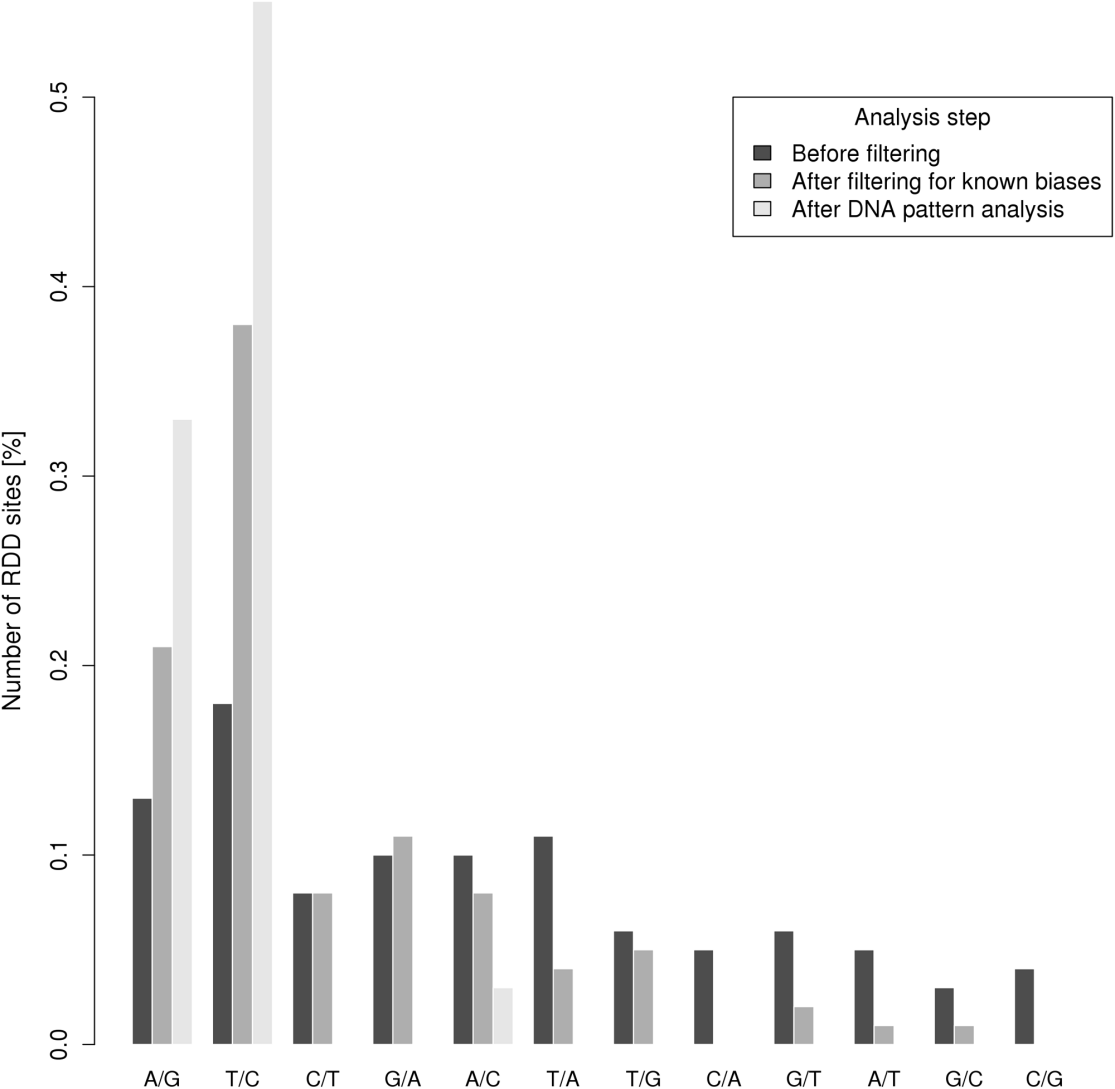
Proportion of base changes of RDD candidates before /after filters.

When comparing our datasets before and after filtering, we observed a clear enrichment in canonical changes throughout successive filters, which was reassuring in terms of results accuracy (Fig 2). Before filtering, all possible base changes were represented, at a frequency ranging from 5% to 20% (Fig 2). Altogether, canonical base changes represented 50% of RDD candidates. After filtering, canonical base changes represented all modifications except one, at position chr6: 29787642. This non-canonical A-to-C position seemed to be the result of a misalignment involving an alternative splice-site. This position was selected for pyrosequencing validation.

Among the canonical modifications, we found only A-to-G or its complement T-to-C modifications, and no C-to-T conversion.

We then characterized the RDD candidates with regards to their putative functional features.

### Functional RDD and tissue expression

**Fig 3:**
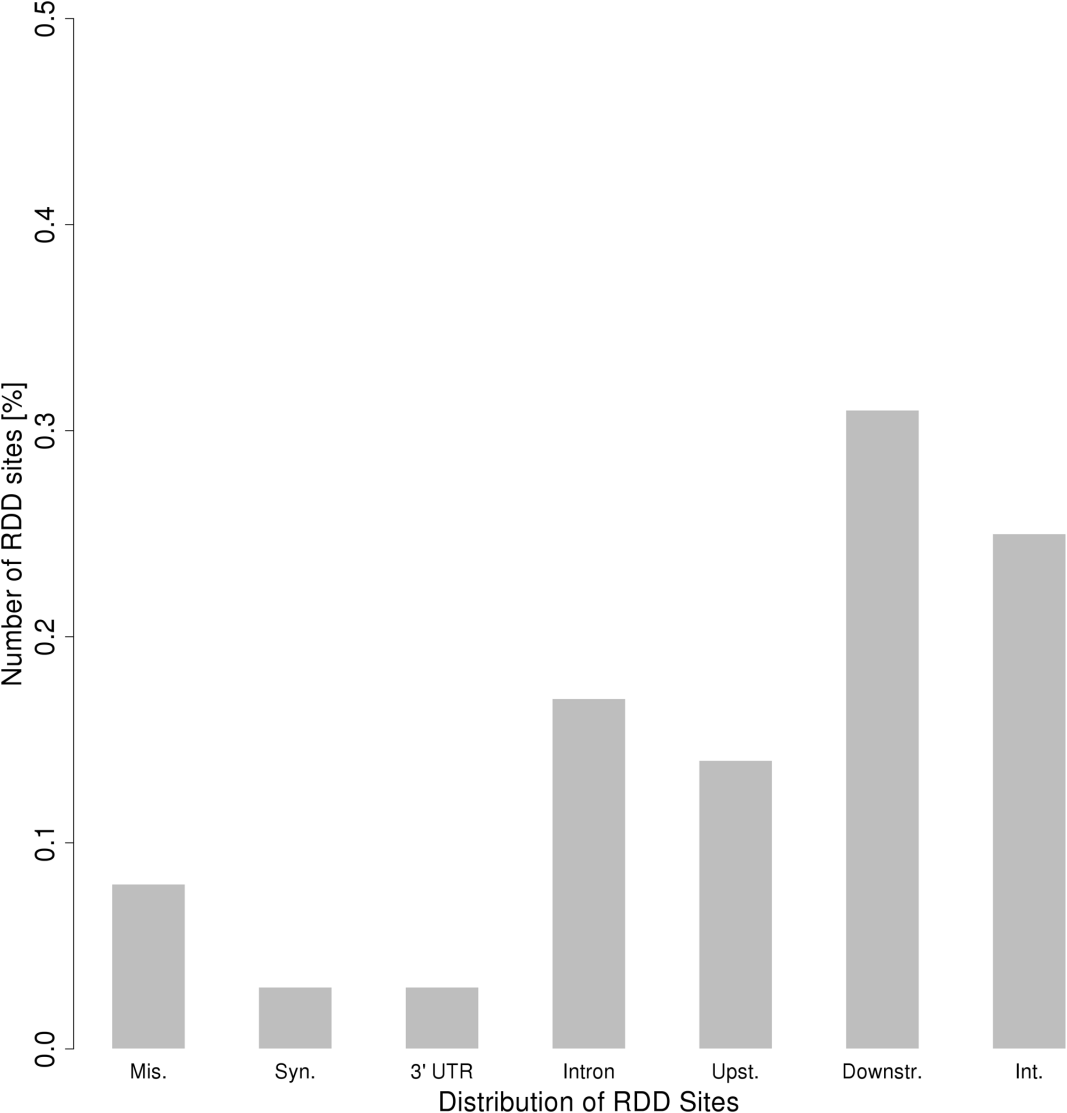
Distribution of RDD sites in genomic features.

Three RDD sites, located on *CYFIP2*, *GRIA2* and *COG3*, were potentially functional, because these changes are non-synonymous, and thus potentially have deleterious effects on the encoded protein. Most of the remaining candidates are located in gene introns, upstream or downstream regions of genes (Table 2, Fig 3).

By using 5 different *in silico* predictors of the amino-acids substitutions’ putative effects, we showed that none of the 3 non-synonymous substitutions was likely to be deleterious (Table 3). These substitutions were localized in highly conserved regions of the proteins (S1 Fig). A striking observation is that the K/E editing site affecting the *CYFIP2* gene changes an amino-acid conserved between all examined vertebrate species into an amino-acid which is coded without editing by the genomic sequence of Ray-finned fishes.

**Table 3:** *In silico* prediction of functional consequence on edited variants.

Variant	SIFT	PolyPhen2	Mut. Ass.	CHASM*	ProSMS
COG3 I632V	tolerated	benign	neutral	0.40 (0.22)	could destabilize
CYFIP2 K320E	tolerated	benign	neutral	0.32 (0.29)	no effect
GRIA2 R764G	tolerated	benign	medium	0.17 (0.49)	no effect

* Computed using Cravat 3.0, functional score close to 1 means functional effect (score p-value)

### Characterization of candidates

**Fig 4:**
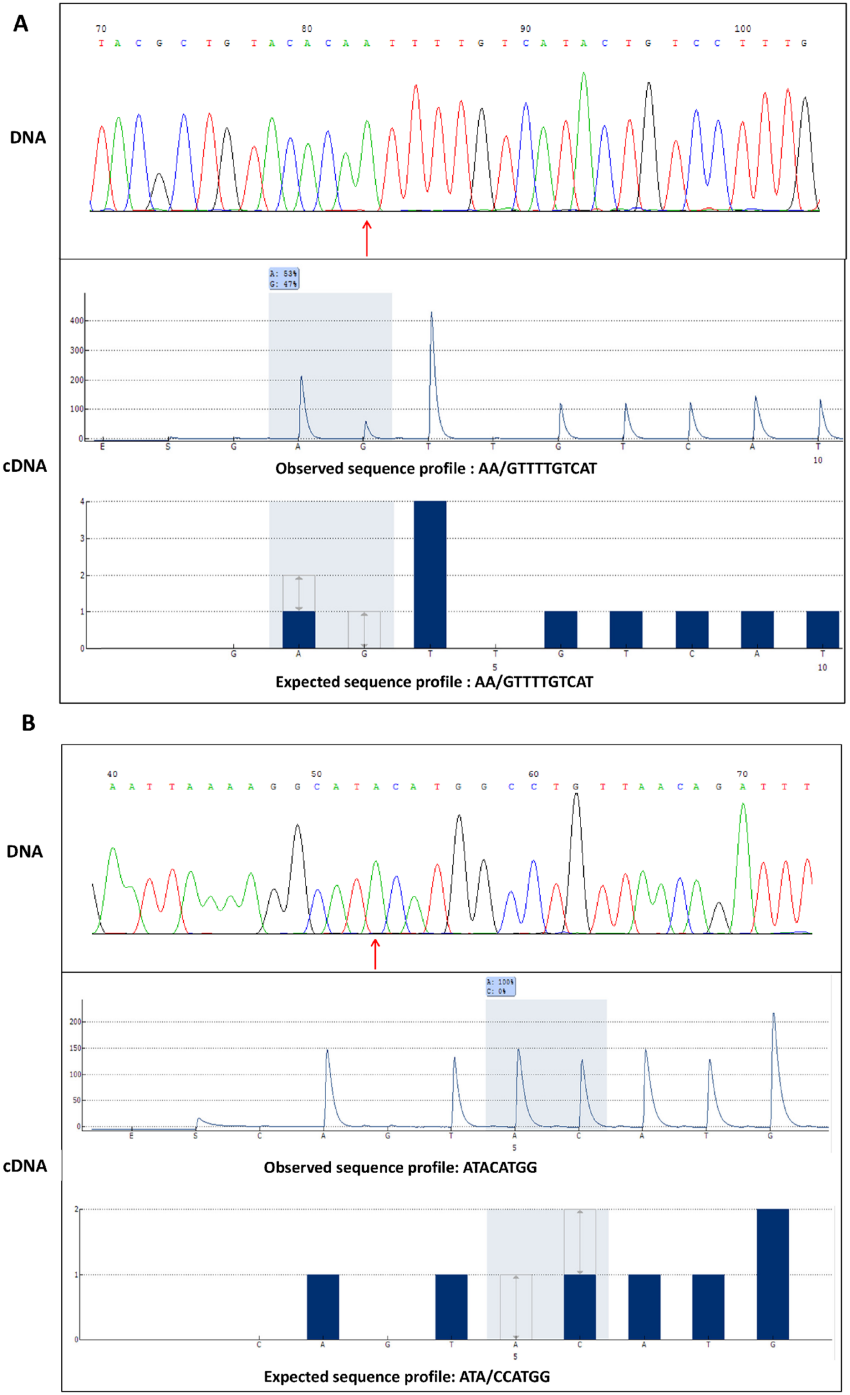
Validation of candidates by Sanger sequencing (DNA) (red arrow) and pyrosequencing (cDNA) (grey). A: Example of a canonical RDD (T-to-C) at position chr2: 86000926. The sequence is in reverse-complement. The RDD status is confirmed by pyrosequencing (A: 53%—G: 47%). B: Example of a non-canonical RDD (A-to-C) at position chr6: 29787642. The alternative nucleotide is not detected (A:100%—C: 0%).

We designed primers for 14 RDD candidates corresponding to 9 genomic regions, comprising missense variants, intron, upstream or downstream regions, intergenic positions, and the remaining non-canonical modification. We first confirmed the homozygous status of the 14 selected RDD sites in DNA by Sanger sequencing. Their RDD status was then tested by pyrosequencing, and 13 RDD candidates were confirmed as edited loci (Fig 4). It is interesting to note that the unique site not validated by pyrosequencing corresponds to the non-canonical RDD candidate.

**Fig 5:**
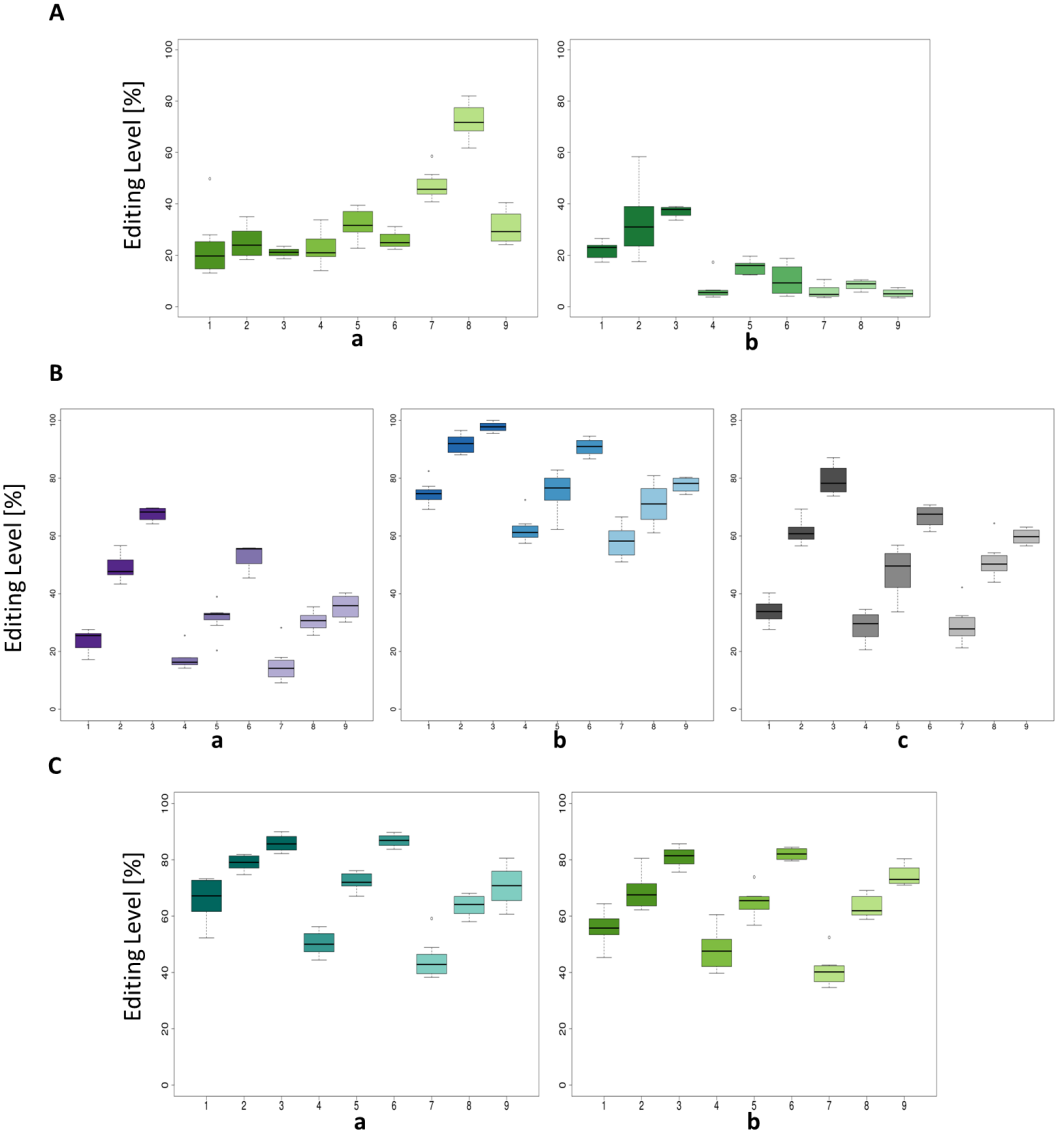
Editing levels observed across tissues and time. A: 2 selected candidates (a: chr1: 167109833; b: chr13: 10717577). B: Cluster 1 candidates (a: chr13: 931843; b: chr13: 931855; c: chr13: 931888). C: Cluster 2 candidates (a: chr2: 86000926; b: chr2: 86001370). On abscissa axis: 1: Embryo stage 4.5 days—Brain, 2: Embryo stage 15 days—Brain, 3: Adult 11 monthsBrain, 4: Embryo stage 4.5 days—Heart, 5: Embryo stage 15 days—Heart, 6: Adult 11 months—Heart, 7: Embryo stage 4.5 days—Liver, 8: Embryo stage 15 days—Liver, 9: Adult 11 months—Liver.

A subset of 7 validated candidates was then tested in the other available tissues: individual heart, brain and liver tissues from three developmental times, comprising the same stage as the original HiSeq samples (day 4.5), an older embryonic stage (day 15), and an adult stage (11 months of age). Among these candidates, three are clustered on chromosome 13 (Fig 5B.abc) and two are clustered on chromosome 2 (Fig 5C.ab). These positions were tested for tissue and stage effects on editing levels (Table 4). Tissue effect and stage effect were significant for all candidates (p-value≤0.05), and an interaction between tissue and stage was also observed for all but one candidate. There was a clear effect of both tissue and stage on the editing level. Interestingly, for 5 candidates out of 7, there was a continuous increase in editing level with age, from about 50% to more than 80%, independently of the tested tissue (Fig 5). In both clustered regions (Fig 5B and 5C), all candidates harbored the same profile and only differed by their editing level. For one candidate, chr1: 167109833 (*COG3* gene, Fig 5Aa), the editing level increased during embryonic development and was less important in adult stage. On chr13: 10717577 (*CYFIP2* gene, Fig 5A.b), editing was mainly present in brain, with an increase of its level over time, and was at low level in other tissues. Interestingly, the editing level was tissue-specific at every developmental time point, and increasing for most of the candidates from liver to brain (Fig 5).

**Table 4:** P-values from an analysis of variance for tissue and stage effect on editing frequency.

Chromosome	Position	Tissue effect	Stage effect	Tissue/Stage Interaction
1	167,109,833	1.29E-19	1.63E-09	1.35E-07
2	86,000,926	1.63E-14	1.13E-21	4.79E-03
2	86,001,370	8.17E-05	2.39E-20	9.94E-02
13	10,717,577	2.08E-17	1.11E-04	2.32E-02
13	931,843	1.44E-16	1.67E-25	1.45E-06
13	931,855	4.02E-15	1.67E-17	0.14
13	931,888	4.57E-08	4.77E-24	0.02

Positions of candidates tested on multiple tissues by pyrosequencing are shown.

## Discussion

Among animals, RNA editing is well described in mammals at a whole genome scale, but similar studies were lacking in other vertebrates like chicken. The goals of this study were to screen the entire chicken transcriptome for editing sites, to characterize this phenomenon at different stages of development and tissues, and to extend the knowledge of its conservation among vertebrates.

To do so, we used DNA-Seq and RNA-Seq technologies, allowing us to screen the whole chicken genome for such events. This approach was used recently in several species to detect RDD [12–15, 26, 27, 42–44]. While a large number of new RDD sites was first described using this approach, in particular in humans with more than 10,000 sites observed [43], these results were then contested [28, 29], suggesting that RNA editing is a limited process when taking into account possible high-throughput sequencing technologies biases. Later studies confirmed this questioning by finding many fewer RDD sites when stringently filtering the dataset [15, 29]. For example, Pickrell and colleagues demonstrated that up to 94% of the 10,210 edited sites highlighted by Li and collaborators [43] were likely to be false positives.

We carefully looked at common analysis pitfalls when detecting RDD sites in our dataset. First, we applied a stringent filter by taking into account only RDD sites observed in at least 2 biological replicates. This filter ensures true biological phenomena are kept, and removes candidates due to individual-dependent artefacts (as specific sequencing errors or somatic mutations putatively not seen in DNA). We then filtered our datasets for known sequence analysis biases as described in the Materials and Methods section. At this step of analysis, we found 112 RDD candidates (Fig 1A, 1B and 1C). Another study filtering its datasets for the same biases, with a more stringent filter concerning the number of biological replicates (at least two thirds of replicates detected as edited) [15], found between 128 (in mouse liver) and 447 (in mouse adipose) RDD candidates at the same filtering step, *i*.*e*. more candidates than our results while their study was limited to the exome. It could constitute an argument in favor of the scarcity of RNA editing in chicken. We chose to be really stringent by keeping only RDD positions with a total absence of the alternative nucleotide in DNA. The final step, eliminating candidates for which the edited pattern was found in the DNA reads, removed 48 candidates.

At the end of the filtering steps, we kept less than 10% of putative RDD sites detected at the beginning of our study, which is similar to results obtained in recent studies, taking into account biases linked to high-throughtput sequencing [15, 17].

Compared with previous studies, a distinguishable feature of our analysis is the search of the "edited" pattern in the DNA reads of candidates highlighted through RNA-Seq. In several cases, while the “edited” RNA reads map to a candidate region, the corresponding DNA reads do not map to the chicken genome: the RNA read can be mapped to a paralogous region due to the splitting of introns, while the original region is either absent from the genome or is carrying too many mismatches between our individuals and the reference sequence. This can be explained by an incomplete genome assembly and / or several regions with assembly errors. Indeed, the chicken genome assembly is still incomplete, especially regarding microchromosomes [45, 46]. The false RDD status of many candidates due to DNA polymorphism in paralogous regions has already been highlighted [14]. A similar observation has been made by Piskol *et al* [30], leading to the conclusion that non-canonical editing sites are likely to be false positive RDDs.

At the end of the filtering steps, the number of RDD candidates was considerably reduced. Our filters were quite stringent, and we may have missed a few real positions. But as we still detected a false positive candidate through experimental validation, this high stringency was surely appropriate.

It appears that in whole chicken embryo, the number of robust editing sites is limited. Nevertheless, some sites known to be edited in chicken were not highlighted in our analysis, either because of reduced editing level, or low level of expression. This could be explained by the use of 4.5 days whole embryos, in comparison with adult tissues in targeted studies.

The proportion of canonical RDD changes increased across filters, which is reassuring about the reliability of the pipeline: only one non-canonical change could be observed, shown not to be a true conversion, due to misalignments along an alternative splice site. The status of this false candidate was confirmed by pyrosequencing.

Every C-to-T conversion detected at the beginning of the pipeline was discarded at a later filter. Moreover, confirming previous studies of RNA editing in chicken, we could not find any editing in APOB transcripts [19]. This is in accordance with the absence of *APOBEC1* in the chicken genome, as this enzyme seems to be required for C-to-U APOB RNA editing in vertebrates [5].

After the detection of RDD sites in the chicken transcriptome, our aim was to further characterize several interesting edited positions.

All the tested candidates were shared between the tissues studied in our analysis, with one candidate presenting a very low level of editing in the liver, whatever the stage (chr13: 10717577, Fig 5Ab). But the editing level varies between the analyzed tissues and ages, confirming in our chicken model that RNA editing varies across times and tissues.

Interestingly, RNA editing levels change over time. As generally observed in mammals, with few exceptions [47–50], the A-to-I editing level increased during development, as it is the case for 6 out of 7 tested candidates. Even if the tissue and stage specificity of RNA editing is clear in candidates tested from cluster regions (Fig 5B and 5C), it is even more pronounced for tested candidates highlighted separately (Fig 5A). These time- and tissue-specific phenomena are not only due to the level of expression of ADARs [49, 51] and more work is needed to decipher the spatio-temporal regulation of RNA editing. The low level of editing at embryonic stages in almost all the tested candidates could be explained by a putative importance for adequate embryologic development, as it was hypothesized for the *GRIA2* Q/R site in mammals [49], even if it has to be confirmed.

As previously highlighted, our results confirm that editing at a particular position often comes with editing sites nearby, but no clear functional explanation has been proposed yet [49]. The regional sequence composition and RNA molecule tertiary structure seem to be involved in these clustered editing sites [52, 53].

Another interesting result is that only a few candidates were directly affecting the protein sequence by changing an amino-acid. It has been shown that RNA editing can impact protein function, like modifying ions channels in some tissues [54, 55], or impacting the ligand-binding affinity [56]. Nevertheless, our results show that RNA editing in chicken is more frequently silent, as already observed [57]. More studies should be performed to confirm these results. But a significant number of candidates are located in non-coding parts of the chicken genome, at least given the current state of the annotation. As in a previous study in human Alu regions [52], we observed a high number of edited sites in introns. Even if our data come from polyA+ RNAs, these sites may correspond to editing in pre-mRNAs. But they may be part of non-coding RNAs too, where editing has been discovered in several species, and the biological significance of which is still largely unknown [57, 58].

We highlighted 3 candidates that were previously described as edited in mammals, one K/E substitution already observed in the *CYPIF2* gene [21], one I/V conversion located in the *COG3* gene [16, 17, 59]—previously described in human, mouse and rat—and the R/G site in the *GRIA2* gene [60], which means that these editing events are not restricted to mammals and appeared before the Sauropsid-Synapsid divergence. Possible implications of an altered editing efficiency at the R/G site in *GRIA2* in mental disorders in human and mouse were recently observed [61]. Concerning the *COG3* editing site, no functional implication is documented at this time, but as underlined in another study [16], the conservation of this site both in mammals and in a broader way in vertebrates implies a putative functional role. Similarly, the functional significance of the conserved *CYFIP2* K/E editing, which is higher in brain than in other tissues in human, is not known, but may be implicated in apoptosis [48].

The editing sites are located in regions highly conserved between vertebrates (S1 Fig). Interestingly, the modification observed in the *CYFIP2* gene results in a conversion from a glutamic acid to a lysine. This amino-acid is only present in the Ray-finned fishes, and is shared by all of them (http://www.ensembl.org/index.html). The other species for which the homologous *CYFIP2* sequence is available are all K-coding at this position, which asks the question of the functionality of this E residue, only present in fishes as a chromosomal codon, but resulting from an editing phenomenon in several vertebrate species, including chicken.

The number of editing sites conserved among vertebrates seems to be very small, and this may be the signature of their functional importance. In this respect an extensive study recently identified only 59 evolutionarily selected sites among mammals [62].

## Conclusions

This study constitutes, to our knowledge, the first whole genome screening of RNA editing in chicken. By using a stringent pipeline, we focused on reliable RNA editing events and thus removed most putative false positives, a big pitfall in RNA editing discovery by high-throughput sequencing. Our pipeline predicts reliable RNA editing sites, avoiding biases encountered when using NGS data, and most of the tested sites are confirmed through an independent validation method. This whole genome analysis shows that the A-to-I editing mechanism may be the only one present in chicken. Furthermore, we show strong tissue and stage effects on editing level with a tendency to increase with age. Several edited loci are conserved between chicken and other vertebrate species, including human, which indicates that, while RNA editing arose long ago in the evolution, some particular nucleotides from a few genes are subject to RNA editing. This conservation is probably linked to the molecular mechanisms involved, but more deeply questions the functionality of editing at these specific loci. Even if the extent to which RNA is edited is more and more fully characterized, a huge effort to discover the putative functionality of this phenomenon is still needed.

## Supporting Information

S1 Fig Alignment of protein sequence from different species. Multi-species alignments were performed through the Muscle program in the PhyleasProg pipeline (phyleasprog.inra.fr), from reference protein sequences of fully sequenced genomes from Ensembl (www.ensembl.org). The red arrows show the amino acid affected by the editing conversion. The overall conservation between all species is depicted under each multi-alignment. A. COG3 (I—>V) B. GRIA2 (R—>G) C. CYFIP2 (K—>E).
(TIF)

S1 Table Sequencing primers. [Btn] Biotin on 5' end for pyrosequencing only.
(XLSX)

## References

[1] The ENCODE Project Consortium. An integrated encyclopedia of DNA elements in the human genome. Nature. 2012;489(7414):57–74. 10.1038/nature11247 22955616 PMC3439153

[2] Benne R, Van den Burg J, Brakenhoff JP, Sloof P, Van Boom JH, Tromp MC. Major transcript of the frameshifted coxII gene from trypanosome mitochondria contains four nucleotides that are not encoded in the DNA. Cell. 1986;46(6):819–26. . 3019552 10.1016/0092-8674(86)90063-2

[3] Knoop V. When you can't trust the DNA: RNA editing changes transcript sequences. Cell Mol Life Sci. 2011;68(4):567–86. 10.1007/s00018-010-0538-9 20938709 PMC11114842

[4] Bass BL. RNA editing by adenosine deaminases that act on RNA. Annu Rev Biochem. 2002;71:817–46. . 12045112 10.1146/annurev.biochem.71.110601.135501 PMC1823043

[5] Gott JM, Emeson RB. Functions and mechanisms of RNA editing. Annu Rev Genet. 2000;34:499–531. . 11092837 10.1146/annurev.genet.34.1.499

[6] Lee JH, Ang JK, Xiao X. Analysis and design of RNA sequencing experiments for identifying RNA editing and other single-nucleotide variants. Rna. 2013;19(6):725–32. 10.1261/rna.037903.112 23598527 PMC3683905

[7] Blanc V, Davidson NO. C-to-U RNA Editing: Mechanisms Leading to Genetic Diversity. J Biol Chem. 2003;278(3):1395–8. 10.1074/jbc.R200024200 12446660

[8] Lau PP, Xiong WJ, Zhu HJ, Chen SH, Chan L. Apolipoprotein B mRNA editing is an intranuclear event that occurs posttranscriptionally coincident with splicing and polyadenylation. J Biol Chem. 1991;266(30):20550–4. 1939106

[9] Gray MW. Evolutionary origin of RNA editing. Biochemistry. 2012;51(26):5235–42. . 22708551 10.1021/bi300419r

[10] Gray MW, Lukes J, Archibald JM, Keeling PJ, Doolittle WF. Cell biology. Irremediable complexity? Science. 2010;330(6006):920–1. 10.1126/science.1198594 21071654

[11] Speijer D. Does constructive neutral evolution play an important role in the origin of cellular complexity? Making sense of the origins and uses of biological complexity. Bioessays. 2011;33(5):344–9. 10.1002/bies.201100010 21381061

[12] Park E, Williams B, Wold BJ, Mortazavi A. RNA editing in the human ENCODE RNA-seq data. Genome Res. 2012;22(9):1626–33. 10.1101/gr.134957.111 22955975 PMC3431480

[13] Ramaswami G, Lin W, Piskol R, Tan MH, Davis C, Li JB. Accurate identification of human Alu and non-Alu RNA editing sites. Nat Methods. 2012;9(6):579–81. 10.1038/nmeth.1982 22484847 PMC3662811

[14] Schrider DR, Gout JF, Hahn MW. Very few RNA and DNA sequence differences in the human transcriptome. PLoS One. 2011;6(10):e25842 10.1371/journal.pone.0025842 22022455 PMC3192132

[15] Lagarrigue S, Hormozdiari F, Martin LJ, Lecerf F, Hasin Y, Rau C, et al Limited RNA editing in exons of mouse liver and adipose. Genetics. 2013;193(4):1107–15. 10.1534/genetics.112.149054 23410828 PMC3606090

[16] Holmes AP, Wood SH, Merry BJ, de Magalhaes JP. A-to-I RNA editing does not change with age in the healthy male rat brain. Biogerontology. 2013;14(4):395–400. 10.1007/s10522-013-9433-8 23708854 PMC3739863

[17] Danecek P, Nellaker C, McIntyre RE, Buendia-Buendia JE, Bumpstead S, Ponting CP, et al High levels of RNA-editing site conservation amongst 15 laboratory mouse strains. Genome Biol. 2012;13(4):26 10.1186/gb-2012-13-4-r26 22524474 PMC3446300

[18] Maas S, Godfried Sie CP, Stoev I, Dupuis DE, Latona J, Porman AM, et al Genome-wide evaluation and discovery of vertebrate A-to-I RNA editing sites. Biochem Biophys Res Commun. 2011;412(3):407–12. 10.1016/j.bbrc.2011.07.075 21835166

[19] Teng B, Davidson NO. Evolution of intestinal apolipoprotein B mRNA editing. Chicken apolipoprotein B mRNA is not edited, but chicken enterocytes contain in vitro editing enhancement factor(s). J Biol Chem. 1992;267(29):21265–72. . 1400437

[20] Severi F, Chicca A, Conticello SG. Analysis of reptilian APOBEC1 suggests that RNA editing may not be its ancestral function. Mol Biol Evol. 2011;28(3):1125–9. 10.1093/molbev/msq338 21172829

[21] Levanon EY, Hallegger M, Kinar Y, Shemesh R, Djinovic-Carugo K, Rechavi G, et al Evolutionarily conserved human targets of adenosine to inosine RNA editing. Nucleic Acids Res. 2005;33(4):1162–8. . 15731336 10.1093/nar/gki239 PMC549564

[22] Irimia M, Denuc A, Ferran JL, Pernaute B, Puelles L, Roy SW, et al Evolutionarily conserved A-to-I editing increases protein stability of the alternative splicing factor Nova1. RNA Biol. 2012;9(1):12–21. 10.4161/rna.9.1.18387 22258141

[23] Daniel C, Wahlstedt H, Ohlson J, Björk P, Öhman M. Adenosine-to-Inosine RNA Editing Affects Trafficking of the γ-Aminobutyric Acid Type A (GABAA) Receptor. J Biol Chem. 2011;286(3):2031–40. 10.1074/jbc.M110.130096 21030585 PMC3023500

[24] Ring H, Boije H, Daniel C, Ohlson J, Ohman M, Hallbook F. Increased A-to-I RNA editing of the transcript for GABAA receptor subunit alpha3 during chick retinal development. Vis Neurosci. 2010;27(5–6):149–57. 10.1017/S0952523810000234 20843408

[25] Wang Y, Ghaffari N, Johnson CD, Braga-Neto UM, Wang H, Chen R, et al Evaluation of the coverage and depth of transcriptome by RNA-Seq in chickens. BMC Bioinformatics. 2011;12 Suppl 10:S5 10.1186/1471-2105-12-S10-S5 22165852 PMC3236848

[26] Peng Z, Cheng Y, Tan BC, Kang L, Tian Z, Zhu Y, et al Comprehensive analysis of RNA-Seq data reveals extensive RNA editing in a human transcriptome. Nat Biotechnol. 2012;30(3):253–60. 10.1038/nbt.2122 22327324

[27] Bahn JH, Lee JH, Li G, Greer C, Peng G, Xiao X. Accurate identification of A-to-I RNA editing in human by transcriptome sequencing. Genome Res. 2012;22(1):142–50. 10.1101/gr.124107.111 21960545 PMC3246201

[28] Kleinman CL, Majewski J. Comment on "Widespread RNA and DNA sequence differences in the human transcriptome". Science. 2012;335(6074):1302; author reply 10.1126/science.1210624 22422962

[29] Pickrell JK, Gilad Y, Pritchard JK. Comment on "Widespread RNA and DNA sequence differences in the human transcriptome". Science. 2012;335(6074):1302; author reply 10.1126/science.1210624 22422963 PMC5207799

[30] Piskol R, Peng Z, Wang J, Li JB. Lack of evidence for existence of noncanonical RNA editing. Nat Biotechnol. 2013;31(1):19–20. 10.1038/nbt.2472 . 23302925

[31] Lin W, Piskol R, Tan MH, Li JB. Comment on “Widespread RNA and DNA Sequence Differences in the Human Transcriptome”. Science. 2012;335(6074):1302 10.1126/science.1210624 22422962

[32] Chen JY, Peng Z, Zhang R, Yang XZ, Tan BC, Fang H, et al RNA editome in rhesus macaque shaped by purifying selection. PLoS genetics. 2014;10(4):e1004274 10.1371/journal.pgen.1004274 24722121 PMC3983040

[33] Frésard L, Leroux S, Servin B, Gourichon D, Dehais P, Cristobal MS, et al Transcriptome-wide investigation of genomic imprinting in chicken. Nucleic Acids Res. 2014;42(6):3768–82. 10.1093/nar/gkt1390 24452801 PMC3973300

[34] Bumstead N, Barrow PA. Genetics of resistance to Salmonella typhimurium in newly hatched chicks. Br Poult Sci. 1988;29(3):521–9. . 3066449 10.1080/00071668808417078

[35] Bordas A, Tixier-Boichard M, Merat P. Direct and correlated responses to divergent selection for residual food intake in Rhode Island Red laying hens. Br Poult Sci. 1992;33(4):741–54. 1393669 10.1080/00071669208417515

[36] Li H, Handsaker B, Wysoker A, Fennell T, Ruan J, Homer N, et al The Sequence Alignment/Map format and SAMtools. Bioinformatics. 2009;25(16):2078–9. 10.1093/bioinformatics/btp352 19505943 PMC2723002

[37] Hansen KD, Brenner SE, Dudoit S. Biases in Illumina transcriptome sequencing caused by random hexamer priming. Nucleic Acids Res. 2010;38(12):e131 10.1093/nar/gkq224 20395217 PMC2896536

[38] Roberts A, Trapnell C, Donaghey J, Rinn JL, Pachter L. Improving RNA-Seq expression estimates by correcting for fragment bias. Genome Biol. 2011;12(3):R22 10.1186/gb-2011-12-3-r22 21410973 PMC3129672

[39] Dohm JC, Lottaz C, Borodina T, Himmelbauer H. Substantial biases in ultra-short read data sets from high-throughput DNA sequencing. Nucleic Acids Res. 2008;36(16):e105 10.1093/nar/gkn425 18660515 PMC2532726

[40] McLaren W, Pritchard B, Rios D, Chen Y, Flicek P, Cunningham F. Deriving the consequences of genomic variants with the Ensembl API and SNP Effect Predictor. Bioinformatics. 2010;26(16):2069–70. 10.1093/bioinformatics/btq330 20562413 PMC2916720

[41] Olson SA. EMBOSS opens up sequence analysis. European Molecular Biology Open Software Suite. Briefings in bioinformatics. 2002;3(1):87–91. . 12002227 10.1093/bib/3.1.87

[42] Bazak L, Haviv A, Barak M, Jacob-Hirsch J, Deng P, Zhang R, et al A-to-I RNA editing occurs at over a hundred million genomic sites, located in a majority of human genes. Genome Res. 2014;24(3):365–76. 10.1101/gr.164749.113 24347612 PMC3941102

[43] Li M, Wang IX, Li Y, Bruzel A, Richards AL, Toung JM, et al Widespread RNA and DNA sequence differences in the human transcriptome. Science. 2011;333(6038):53–8. 10.1126/science.1207018 21596952 PMC3204392

[44] Picardi E, Horner DS, Chiara M, Schiavon R, Valle G, Pesole G. Large-scale detection and analysis of RNA editing in grape mtDNA by RNA deep-sequencing. Nucleic Acids Res. 2010;38(14):4755–67. 10.1093/nar/gkq202 20385587 PMC2919710

[45] Groenen MA, Megens HJ, Zare Y, Warren WC, Hillier LW, Crooijmans RP, et al The development and characterization of a 60K SNP chip for chicken. BMC genomics. 2011;12(1):274 10.1186/1471-2164-12-274 21627800 PMC3117858

[46] International Chicken Genome Sequencing C. Sequence and comparative analysis of the chicken genome provide unique perspectives on vertebrate evolution. Nature. 2004;432(7018):695–716. 10.1038/nature03154 . 15592404

[47] Enstero M, Daniel C, Wahlstedt H, Major F, Ohman M. Recognition and coupling of A-to-I edited sites are determined by the tertiary structure of the RNA. Nucleic Acids Res. 2009;37(20):6916–26. 10.1093/nar/gkp731 19740768 PMC2777444

[48] Shtrichman R, Germanguz I, Mandel R, Ziskind A, Nahor I, Safran M, et al Altered A-to-I RNA editing in human embryogenesis. PLoS One. 2012;7(7):e41576 10.1371/journal.pone.0041576 22859999 PMC3409221

[49] Veno MT, Bramsen JB, Bendixen C, Panitz F, Holm IE, Ohman M, et al Spatio-temporal regulation of ADAR editing during development in porcine neural tissues. RNA Biol. 2012;9(8):1054–65. 10.4161/rna.21082 22858680 PMC3551860

[50] Wahlstedt H, Daniel C, Enstero M, Ohman M. Large-scale mRNA sequencing determines global regulation of RNA editing during brain development. Genome Res. 2009;19(6):978–86. 10.1101/gr.089409.108 19420382 PMC2694479

[51] Garncarz W, Tariq A, Handl C, Pusch O, Jantsch MF. A high-throughput screen to identify enhancers of ADAR-mediated RNA-editing. RNA Biol. 2013;10(2):192–204. 10.4161/rna.23208 23353575 PMC3594278

[52] Athanasiadis A, Rich A, Maas S. Widespread A-to-I RNA editing of Alu-containing mRNAs in the human transcriptome. PLoS biology. 2004;2(12):e391 10.1371/journal.pbio.0020391 15534692 PMC526178

[53] Tian N, Yang Y, Sachsenmaier N, Muggenhumer D, Bi J, Waldsich C, et al A structural determinant required for RNA editing. Nucleic Acids Res. 2011;39(13):5669–81. 10.1093/nar/gkr144 21427087 PMC3141254

[54] Seeburg PH, Hartner J. Regulation of ion channel/neurotransmitter receptor function by RNA editing. Current Opinion in Neurobiology. 2003;13(3):279–83. 10.1016/S0959-4388(03)00062-X 12850211

[55] Song W, Liu Z, Tan J, Nomura Y, Dong K. RNA Editing Generates Tissue-specific Sodium Channels with Distinct Gating Properties. J Biol Chem. 2004;279(31):32554–61. 10.1074/jbc.M402392200 15136570 PMC3066004

[56] Wang Q, O'Brien PJ, Chen C-X, Cho D-SC, Murray JM, Nishikura K. Altered G Protein-Coupling Functions of RNA Editing Isoform and Splicing Variant Serotonin2C Receptors. J Neurochem. 2000;74(3):1290–300. 10.1046/j.1471-4159.2000.741290.x 10693963

[57] Nishikura K. Functions and regulation of RNA editing by ADAR deaminases. Annu Rev Biochem. 2010;79:321–49. 10.1146/annurev-biochem-060208-105251 20192758 PMC2953425

[58] Savva YA, Reenan RA. Identification of evolutionarily meaningful information within the mammalian RNA editing landscape. Genome Biol. 2014;15(1):103 10.1186/gb4157 24468094 PMC4053966

[59] Shah SP, Morin RD, Khattra J, Prentice L, Pugh T, Burleigh A, et al Mutational evolution in a lobular breast tumour profiled at single nucleotide resolution. Nature. 2009;461(7265):809–13. http://www.nature.com/nature/journal/v461/n7265/suppinfo/nature08489_S1.html. 10.1038/nature08489 19812674

[60] Yang JH, Sklar P, Axel R, Maniatis T. Purification and characterization of a human RNA adenosine deaminase for glutamate receptor B pre-mRNA editing. Proceedings of the National Academy of Sciences of the United States of America. 1997;94(9):4354–9. 9113993 10.1073/pnas.94.9.4354 PMC20726

[61] Kubota-Sakashita M, Iwamoto K, Bundo M, Kato T. A role of ADAR2 and RNA editing of glutamate receptors in mood disorders and schizophrenia. Molecular brain. 2014;7:5 10.1186/1756-6606-7-5 24443933 PMC3902024

[62] Pinto Y, Cohen HY, Levanon EY. Mammalian conserved ADAR targets comprise only a small fragment of the human editosome. Genome Biol. 2014;15(1):R5 10.1186/gb-2014-15-1-r5 24393560 PMC4053846

