# Supplementary figures and images for: Genome-Wide Characterization of RNA Editing in Chicken Embryos Reveals Common Features among Vertebrates
Source: PLoS One. 2015 May 29;10(5):e0126776. doi: 10.1371/journal.pone.0126776 (PMC4449034; doi:10.1371/journal.pone.0126776)

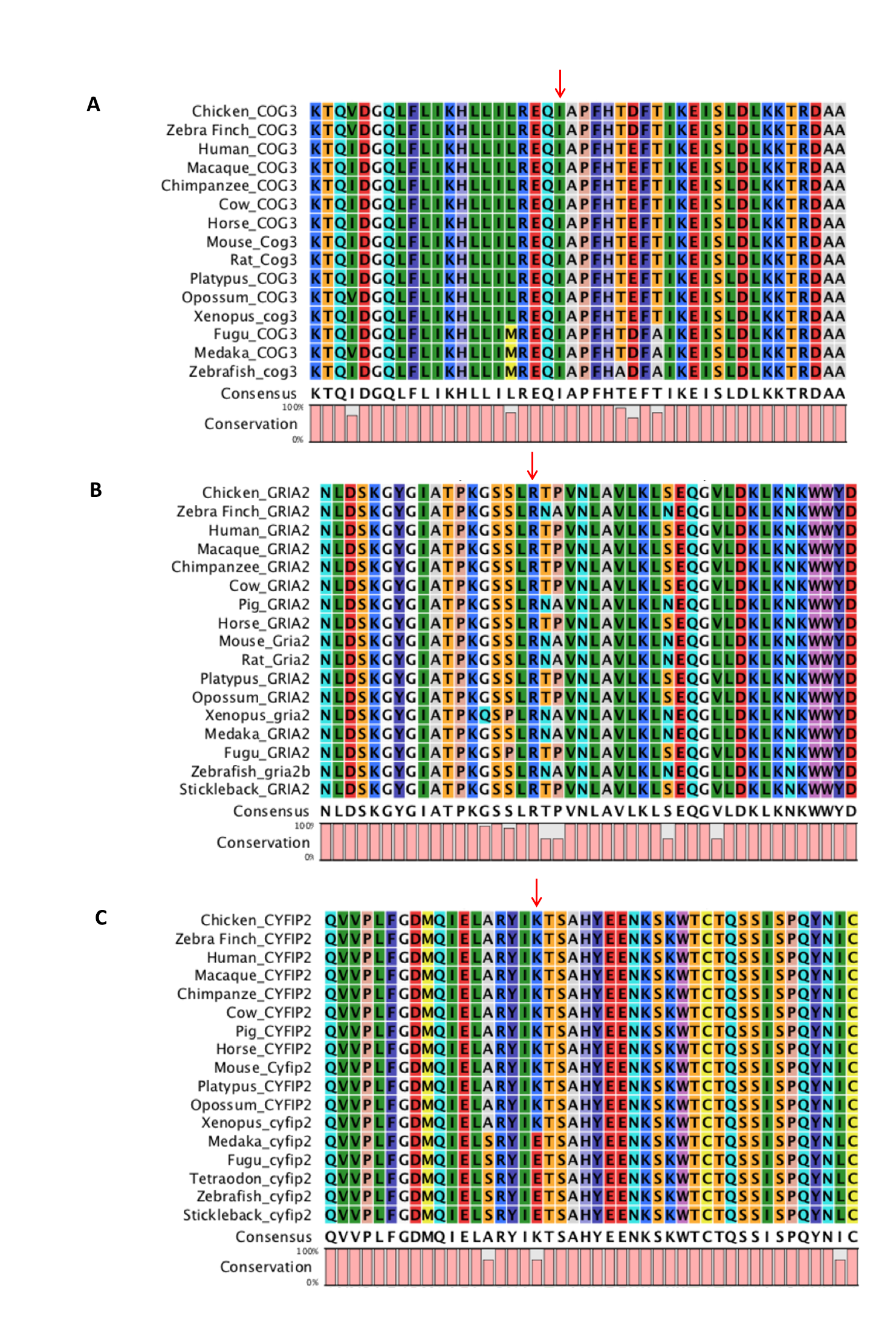

Supplement: S1 Fig — Multi-species alignments were performed through the Muscle program in the PhyleasProg pipeline (phyleasprog.inra.fr), from reference protein sequences of fully sequenced genomes from Ensembl (www.ensembl.org). The red arrows show the amino acid affected by the editing conversion. The overall conservation between all species is depicted under each multi-alignment. A. COG3 (I—>V) B. GRIA2 (R—>G) C. CYFIP2 (K—>E). (TIF) [file pone.0126776.s001.tif]
